# Chicago Society of Physicians and Surgeons

**Published:** 1872-06-01

**Authors:** 


### CHICAGO SOCIETY OF PHYSICIANS AND SURGEONS.

At the first regular meeting of the "Chicago Society of Physicians and Surgeons," on May 1st, Dr. Trimble being temporary Chairman, the Constitution and By-Laws, as presented by the Committee appointed to draft the same, were formally accepted. The Constitution providing that its membership should consist exclusively of "graduates of some respectable, regular school of medicine possessing a good moral and professional reputation," only such were allowed to unite for the object contemplated. The Committee appointed to correspond with the Chicago Medical Society, reported by presenting the letter forwarded to that body, informing them of the organization and standard of membership of the Society of Physicians and Surgeons, their adhesion to the Code of Ethics of the American Medical Association, the object of the institution of the Society, and asking for sympathy and encouragement. The report of the committee was accepted. Dr. A. R. Jackson then read a report of the removal of a fibroid cystic tumor from a female, accompanying it with specimen of the same. The following officers were then elected for the current year:

Dr. D. B. Trimble, *President.*

Dr. W. C. Lyman, *Vice-President.*

Dr. J. N. Hyde, *Secretary and Treasurer.*

Drs. McKennan, Emmons and Jackson, *Censors.*

The second regular meeting of the Society was held in the parlor of the Chicago Orphan Asylum on Michigan avenue, May 27. The minutes of the preceding meeting were approved. Drs. D. D. Thompson, of Geneva, 1844, and Dr. P. S. Hayes, Rush, 1872, were proposed in writing for membership and their names referred to the Censors. Dr. Lyman read a report of cases of cerebro-spinal meningitis, which was subsequently discussed by the Society. Some pathological photographs were then exhibited to the Society by Mr. John L. Gihon of Philadelphia, through the graphoscope, and received the thanks of the Society for the same.

Dr. Hyde gave notice of the reading of a paper on "Syphilitic lesions of the Viscera," after the continuation of the discussion on cerebro-spinal meningitis, which was deferred to the next meeting, and the Society adjourned. J. N. Hyde, M. D., *Secretary.*

Doctors in Russia.—We find from an official report that last year there were 10,000 doctors in Russia. Of this number 6,113 held public appointments, and 4,686 confined themselves to private practice. This gives only about one doctor to 7,182 of the inhabitants, basing the calculation on the most recent returns of the population. We know of districts in Russia where there is no doctor within less than a day's journey, and in some parts it is probable that there is still greater difficulty in obtaining medical aid.—*Doctor.*

